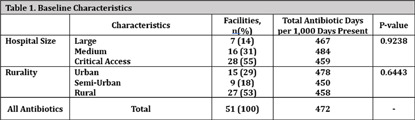# Analyzing Antibiotic Usage Rates Reported to NHSN by Nebraska Hospitals: Insights by Hospital Size and Rurality

**DOI:** 10.1017/ash.2025.241

**Published:** 2025-09-24

**Authors:** Rabia Syed, Jenna Preusker, Juan Teran Plasencia, Trevor Van Schooneveld, Scott Bergman, Danny Schroeder, M. Salman Ashraf

**Affiliations:** 1 Nebraska Department of Health & Human Services; 2 Nebraska Medicine/Nebraska DHHS; 3 UNMC; 4 University of Nebraska Medical Center; 5 University of Nebraska Medical Center; 6 Nebraska Medicine Nebraska ASAP; 7 University of Nebraska Medical Center

## Abstract

**Background:** State-level hospital comparative antibiotic usage rates can highlight opportunities for interventions to optimize antimicrobial stewardship (AS). We sought to characterize antibiotic usage rates for Nebraska hospitals stratified by hospital size and rurality. **Methods:** NHSN antibiotic use (AU) data reported from September 2023 to August 2024 was extracted. Hospitals reporting adult data for any antibiotics of interest were included in analysis. Data from all units reported by the hospital were included. Hospital sizes were categorized by number of beds reported to NHSN: critical access (≤25 beds), medium (26-150 beds), and large (>150 beds). Rurality was defined using the USDA rural urban commuting area codes: urban (1-3), semi-urban (4-6), and rural (7-10). AU rate was calculated using antimicrobial days of therapy over 1,000 days present. Pooled AU rates were used to provide a state rate by covariates and antibiotics. Descriptive statistics were used to describe prescribing patterns. A negative binomial regression model was used to understand the effect of hospital size and rurality on rate and one-way ANOVA to test significance. **Results:** AU data was analyzed for 51 facilities including 7 large hospitals (14%), 16 medium-sized hospitals (31%), and 28 critical access hospitals (55%). Of these, 27 facilities (53%) were located in rural and 9 (18%) in semiurban areas (Table 1). The top 5 antibiotics used were cefazolin, ceftriaxone, piperacillin/tazobactam, vancomycin, and cefepime (Figure 1). Although, there were no significant variations in total AU based on hospital size and rurality, some significant differences were noted when broken down by specific antibiotics (Figures 2 and 3). Critical access hospitals reported 1.8 times higher AU rate for ceftriaxone [95% CI: 1.2, 2.7], 2.2 times the rate for fluoroquinolones [95% CI: 1.3, 3.7], and 2.3 times the rate for azithromycin [95% CI: 1.4, 3.7], compared to large hospitals. Similarly compared to urban hospitals, rural hospitals reported 1.9 times higher AU rate for ceftriaxone [95% CI: 1.4, 2.5], 2.3 times the rate for fluoroquinolones [95% CI: 1.6, 3.4], and 2.2 times the rate for azithromycin [95% CI: 1.6, 3.2]. No significant difference was noted in the use of any antibiotics when comparing semiurban to urban and medium to large size hospitals. **Conclusions:** Significant variation exists in use of some antibiotics based on the hospital size and rurality. NHSN AU data can be leveraged to identify potential AS targets across various hospital settings.

**Figure:**
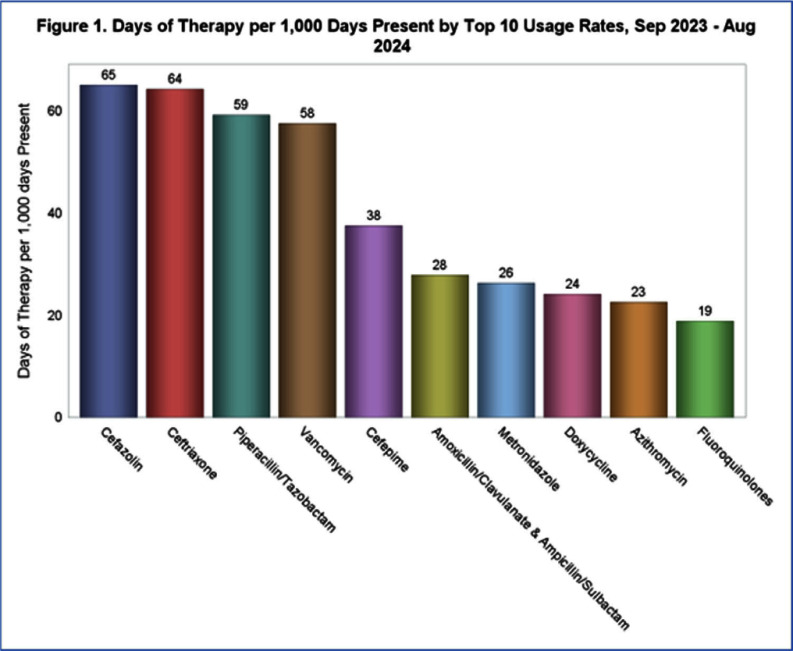

**Figure:**
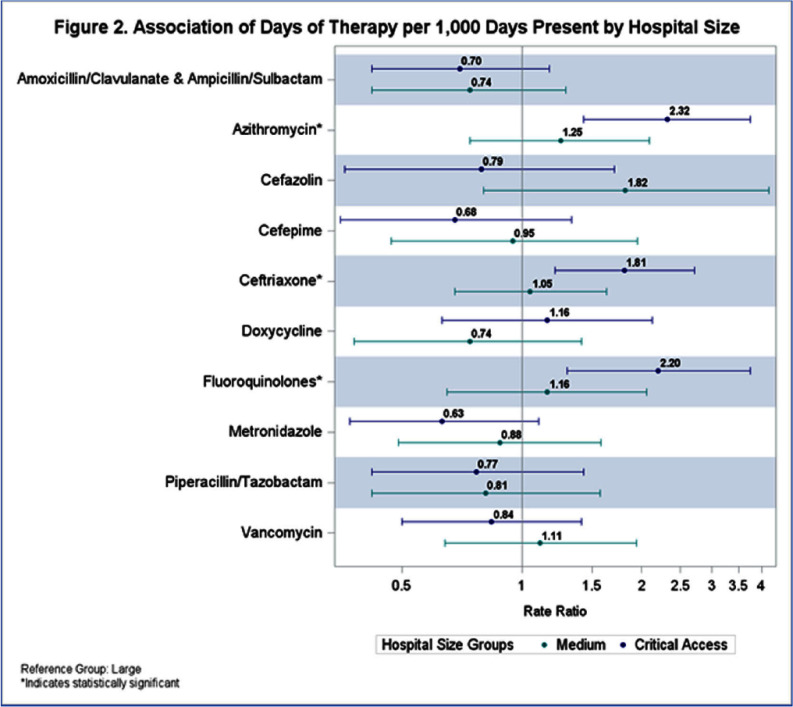

**Figure:**
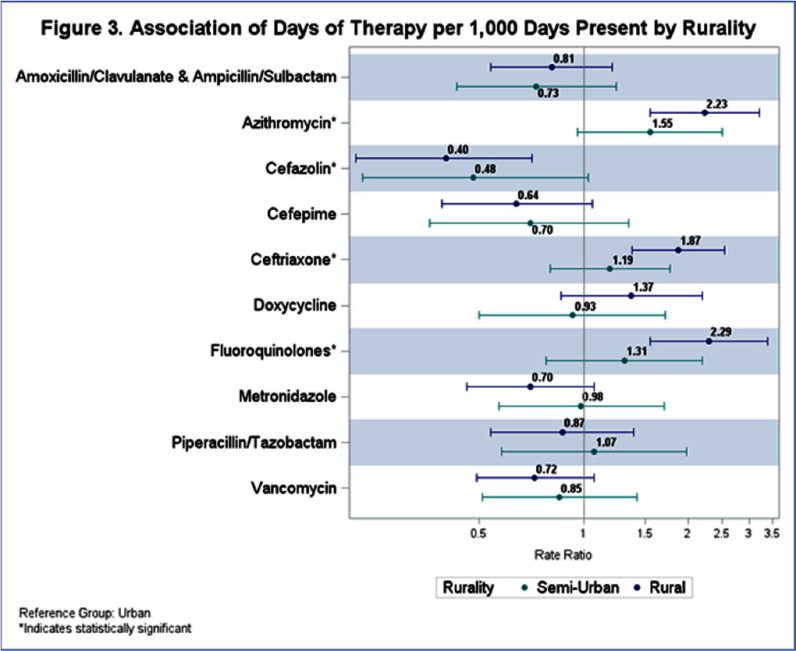

**Figure:**